# Detection method of absence seizures based on Resnet and bidirectional GRU

**DOI:** 10.1186/s42494-022-00117-w

**Published:** 2023-03-01

**Authors:** Lijun Li, Hengxing Zhang, Xiaomei Liu, Jie Li, Lei Li, Dan Liu, Jieqing Min, Ping Zhu, Huan Xia, Shangkun Wang, Li Wang

**Affiliations:** 1 Kunming children‘s hospital, Kunming, 650000 China; 2 Zhengzhou Zoneyet Technology Corp.,Ltd, Zhengzhou, 450000 China

**Keywords:** Resnet, Epilepsy, GRU, Electroencephalogram

## Abstract

**Background:**

Epilepsy is a common chronic neurological disease. Its repeated seizure attacks have a great negative impact on patients’ physical and mental health. The diagnosis of epilepsy mainly depends on electroencephalogram (EEG) signals detection and analysis. There are two main EEG signals detection methods for epilepsy. One is the detection based on abnormal waveform, the other is the analysis of EEG signals based on the traditional machine learning. The feature extraction method of the traditional machine learning is difficult to capture the high-dimension information between adjacent sequences.

**Methods:**

In this paper, redundant information was removed from the data by Gaussian filtering, downsampling, and short-time Fourier transform. Convolutional Neural Networks (CNN) was used to extract the high-dimensional features of the preprocessed data, and then Gate Recurrent Unit (GRU) was used to combine the sequence information before and after, to fully integrate the adjacent information EEG signals and improve the accuracy of the model detection.

**Results:**

Four models were designed and compared. The experimental results showed that the prediction model based on deep residual network and bidirectional GRU had the best effect, and the test accuracy of the absence epilepsy test set reached 92%.

**Conclusions:**

The prediction time of the network is only 10 sec when predicting four-hour EEG signals. It can be effectively used in EEG software to provide reference for doctors in EEG analysis and save doctors’ time, which has great practical value.

## Background

Epilepsy is the fourth most common neurological disorder in the world [1], which seriously affects people’s health and brings great inconvenience to patients. According to the statistics from the China Association Against Epilepsy, the prevalence rate of epilepsy in China is about 8.0%, and the annual incidence rate is 28.9 per 100,000 person. Absence epilepsy is the most common epilepsy syndrome. According to the International League Against Epilepsy 2017 classification scheme [2], absence seizures were divided into typical absence seizures (TAS), atypical absence seizures, myoclonic absence seizures, and eyelid myoclonic absence.

TAS is characterized by sudden loss of consciousness, or staring blankly, with a dull expression, unresponsive to the outside world, and generally not falling or dropping objects. The remission rate is as high as 80%. It is of great significance for the detection and timely treatment of typical absence seizures.

Among all bioelectrical signals, the electroencephalogram (EEG) signals can most directly reflect brain activities. It has the characteristics of small amplitude, strong noise, low frequency and strong randomness. The EEG signals of TAS show a generalized 3 Hz rhythmic spike-slow complex wave dominated by the former head with explosive symmetrical and synchronous discharges, with the highest amplitude in the frontal and central regions [1]. Some artifact signals incorporated in the process of signal acquisition, such as blink, eye movement, electromyography, electrocardiogram, etc. bring great challenges to the detection of precise seizure time [3–5].

Dietch firstly used Fourier transform to analyze EEG signals. Then researchers used classical signal processing methods such as time-frequency analysis [6] and wavelet transform [7, 8] to analyze EEG signals features. In recent years, with the development and application of computer technology, some modern methods such as Recurrence Plots [9] and artificial neural networks [10] have also been gradually applied to the analysis of EEG signals. Due to the current in the detection device and various artifacts in EEG traditional detection methods have poor robustness for the detection of typical absence seizures in different patients [3–8].

Existing seizure detection methods based on machine learning do not comprehensively consider the correlation features among different channels and cannot fuse the information between adjacent sequences of unified channels, resulting in the inability to accurately detect the time of absence seizures [11–13]. Therefore, this paper proposes a prediction algorithm based on deep residual network and bidirectional Gate Recurrent Unit (GRU). The algorithm extracts the high-dimension information of multi-channel absence through Convolutional Neural Networks (CNN), and extracts the information between adjacent sequences through the bidirectional GRU module, which improves the local epilepsy detection ability and the detection accuracy of the model.

## Methods

### Data collection and pre-processing

The data used in the manuscript was collected in Kunming Children’s Hospital, including a total of 1530 EEG signals of 94 males and 59 females. A total of 8233 segments of typical absence seizure waveform were included. The data distribution of typical absence epilepsy is shown in Table 1.

**Table 1:** The data distribution of typical absence epilepsy

	Age.	Pat.	Num.	Dur. (min)
1	1 ~ 3	106	428	76.69
2	3 ~ 7	509	2232	456.07
3	7 ~ 10	494	3470	766.29
4	10 ~ 15	421	2215	453.33

Age.: Age distribution

Pat.: Number of patients

Num.: Number of TAS seizure

Dur.: Total seizure time

There are many methods for epilepsy preprocessing, including smoothing filtering, Gaussian filtering, Kalman filtering, principal component analysis (PCA), K-L matrix transform, short-time Fourier transform (STFT), wavelet transform (DWT) and so on.

Smoothing filtering is difficult to filter out the prominent burr signal. The feature extraction of the descending dimension of PCA technology is only based on the variance to extract the relevant features, which does not use the class information of the sample and the effect is not very good. Although K-L matrix transformation can achieve minimum distortion, the transformation matrix will be transformed with the information between different EEG signals which lead to a poor robustness. The base wave selection and decomposition of DWT algorithm have a great influence on the frequency analysis of signals.

The data was preprocessed by high-order Butterworth filter, and short-time Fourier transform. The results of the three preprocessing methods were inputed into the model for model training. During the test, multiple models were compared for measurement and comparison. The comparison models included the models of residual network with different depths, and the ordinary convolution model.

### Butterworth filter

Butterworth filter was proposed by Stephen Butterwort in 1930. The frequency response curve in the characteristic pass band is flatten to the maximum extent. Starting with the boundary angular frequency, the amplitude decreases gradually with the increase of angular frequency and tends to be negative infinite.

In order to filter out some irrelevant spectral information and 50 Hz frequency interference, the filter was designed as band-pass filter (BPF). The cutoff frequencies were 0.16 and 35, and the filter order was 12.

### Short-time Fourier transform

STFT solves the problem of poor global time positioning of Fourier transform, which cannot reflect the time positioning of different frequencies. The spectrum of a specific long signal is analyzed by window length, and the spectrum characteristics in a certain time period are analyzed by moving window position. The calculation formula of STFT is as follows:

$$STFT\left(f,t\right)=\int_{-\infty}^{+\infty }x\left(\tau \right)h\left(\tau -t\right){e}^{-i2\pi ft} d\tau$$

Where *x*(*t*)represents a signal in the time domain, *h*(*t*)represents a window function, and the entire time window slides over the original signal as *τ* goes.

The short-time Fourier transform selects a time-frequency localized window function, moves the window function, and calculates the power spectrum at different times, and converts the EEG signals from the time domain to the frequency domain information through the short-time Fourier transform, which is more conducive to learning of information by network models.

The time window represents the temporal fineness of expression at each point after the STFT transition. The frequency window represents the frequency domain range of the matrix generated during the short-time Fourier transform. During STFT transformation, the frequency resolution will show a decrease trend as the time domain window size extend. Therefore, during the training process of the model, the time windows were tested with values of 64, 128, 256, and 512, and the representative times were: 0.75 sec, 1.25 sec, 2.5 sec, and 5 sec. Experimental results showed that the model with the window containing 2.5 sec frequency domain information had the highest detection accuracy.

### Combined analysis

Spectrogram and original wave were used while EEG signals analysis since both frequency and amplitude of EEG signals were significant in EEG diagnosis. The original signal was filtered by butter worth filter before segmented to the same shape of spectrogram by utilizing the same window size and overlap rate. The segmented signal then were concatenated with the spectrogram which consisted the input feature of the Network.

### Network framework

Due to different acquisition equipment and acquisition time, different absence epilepsy data contains different channels. In order to facilitate the feature extraction of the subsequent CNN, only the specific channels information of the data was analyzed, and the related names of the extracted channels were: “fp1”, “fp2”, “f3”, “f4”, “c3”, “c4”, “p3”, “p4”, “o1”, “o2”, “f7”, “f8”, “t3”, “t4”, “t5”, “t6”, “fz”, “cz”, “pz”, with a total of 19 channels.

**Fig. 1:**
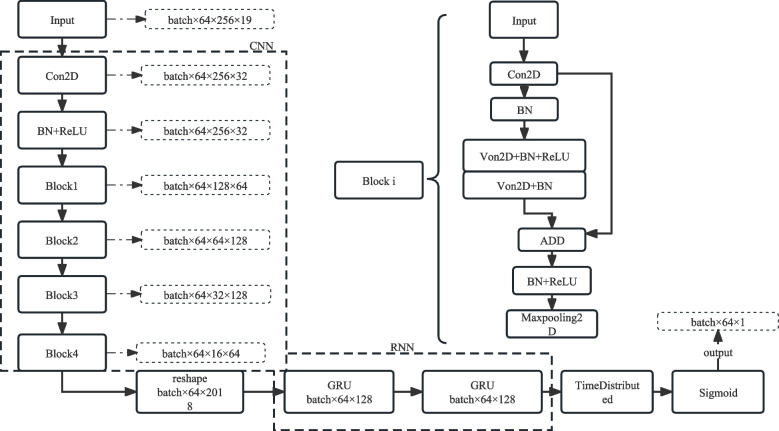
The network framework of CNN and bidirectional GRU

Figure 1 is the network framework of CNN and bidirectional GRU.

### Residual network

CNN has powerful processing ability for input EEG signals. The framework mainly included Convolution layer, Batch Normalization layer, Activation layer and Pooling layer. Convolution operation was to make the number, window size convolution kernel along the data window step by step sliding to do tensor product operation. The activation function solved the linear inseparable problem and increased the expression ability of the neural network. The pooling layer maximized the data in the given window and reduced the operation parameters of the subsequent convolution network.

Batch Normalization prevented exploding gradients and vanishing gradients. This was done by subtracting the mean from the input data and dividing by the variance, so that the data was processed to have a mean of zero and a variance of one. Residual units were implemented in the form of skip layer connections, where the output of the unit was directly added to the input of the unit before activation.

The CNN framework used a residual network framework of four residual blocks. The input feature was (batch * sequence * feature * channel) and the size was (None, 64, 256, 19). Epoch represented the number of times the model learned from the entire input data during the training process of the model on the data. The size of the epoch was related to the degree of diversity of the dataset. The stronger the degree of data diversity, the larger the epoch was set.

Within an epoch, the data was sent to the network, completing a forward calculation and backward propagation process. Batch represented the number of training samples in one learning. Sequence represents the information learned in a batch during model training. Feature represented the information of the time window. When Feature was 256, a vector of 256 dimensions was used to represent the information features in the window.

The detailed network parameters are shown in Table 2.

**Table 2:** The detailed Resnet parameters

layer	The name of network layer	Activation function	Output size	Convolution kernel size	Filters	parameters
	input	–	64 × 256 × 19	–	–	
1	conv2d_1	RELU	64 × 256 × 32	3 × 3	32	5472
2	conv2d_2	–	64 × 256 × 32	3 × 3	32	9216
3	conv2d	–	64 × 256 × 32	1 × 1	32	640
4	Add+RELU	RELU	64 × 256 × 32	–	–	–
5	Max_pooling	–	64 × 128 × 32	–	–	–
6	conv2d_3	–	64 × 128 × 64	1 × 1	64	2112
7	conv2d_4	RELU	64 × 128 × 64	3 × 3	64	18,432
8	conv2d_5	–	64 × 128 × 64	3 × 3	64	36,864
9	Add+RELU	RELU	64 × 128 × 64	–	–	–
10	Max_pooling	–	64 × 64 × 64	–	–	–
11	conv2d_6	–	64 × 64 × 128	1 × 1	128	8320
12	conv2d_7	RELU	64 × 64 × 128	3 × 3	128	73,728
13	conv2d_8	–	64 × 64 × 128	3 × 3	128	147,456
14	Add+RELU	RELU	64 × 64 × 128	–	–	–
15	Max_pooling	–	64 × 32 × 128	–	–	–
16	conv2d_9	–	64 × 32 × 64	1 × 1	128	8256
17	conv2d_10	RELU	64 × 32 × 64	3 × 3	128	73,728
18	conv2d_11	–	64 × 32 × 64	3 × 3	128	36,864
19	Add+RELU	RELU	64 × 32 × 64	–	–	–
20	Max_pooling	–	64 × 16 × 64	–	–	–

### Bidirectional GRU

The RNN network is good at dealing with timing information. However, when the nodes of the neural network are calculated in many stages, the characteristics of the previous longer time are easily covered, forming a long-term dependence problem, and resulting in gradient vanishing and gradient explosion. GRU is a good solution to the large interval dependencies in time series data.

The EEG signals seizure information of absence epilepsy did not exist only in a single channel, nor simply in a certain moment. The network needed to learn and predict the seizures time according to the sequence and the mutual information among the channels. The bidirectional GRU network could extract the information characteristics of frame information before and after fusion learning, and output the seizure time detection results of absence epilepsy EEG signals by analyzing the fusion characteristics of sequence frames. The network unit of GRU is shown in Table 3.

**Table 3:** The detailed network parameters of GRU

layer	The name of network layer	Activation function	Output size	Convolution kernel size	Filters	parameters
	RNN_input	–	64 × 16 × 164	–	–	–
1	Reshape	–	64 × 1024	–	–	–
2	Bidirectional	tanh	64 × 128	0	128	886,272
3	Bidirectional_1	tanh	64 × 128	0	128	18,144
4	Time_distributed	–	64 × 128	–	128	16,512
5	Time_distributed_1	–	64 × 1	–	1	129
6	Output	RELU	64 × 1	–	–	–

### Experimental indicators

The sensitivity, specificity, positive prediction rate and negative prediction rate are used to evaluate the performance of the model. In this paper, positive cases represent the time period when the overlap rate between the detected absence seizure time and the real seizure time is greater than 2 sec, and negative cases represent the time period when the overlap rate between the detected absence seizure time and the real seizure time is less than 2 sec. The evaluation indicators are defined as follows:

1. Sensitivity (true positive rate, TPR): the proportion of identified positive cases in all positive cases is described
  $$TPR=\frac{TP}{TP+ FN}$$
2. Specificity (true negative rate, TNR): the proportion of identified negative cases in all negative cases is described
  $$TNR=\frac{TN}{TN+ FP}$$
3. Positive predictive value (PPV): the proportion of identified positive cases to predicted positive cases is described
  $$PPV=\frac{TP}{TP+ FP}$$
4. Negative predictive value (NPV): the proportion of identified negative cases to predicted negative cases is described
  $$NPV=\frac{TN}{TN+ FN}$$
5. F1: F1 is an indicator used in statistics to measure the accuracy of binary classification models. It is the harmonic mean of model TPR and PPV
  $$F\mathit1=\frac{2\ast TPR\ast PPV}{TPR+PPV}$$

## Results

The main process of this paper included three parts: data preprocessing, CNN high dimension feature extraction, and RNN data analysis. The sampling time of the data was generally from dozens of minutes to 4 h, a total of 1520 sample data, including seizures time accounted for 1.08% of the total time. Since the input data in the training process was of variable length, it was necessary to unbalance the data. A total of 1527 EEGs were used for training and 270 for testing. The sampling frequencies of EEG signals were different. In order to make the characteristic information contained in the signals at the same time be the same, all the training data were down-sampled to 100 Hz by the down-sampling method, and the redundant information of the data in the training process was reduced.

**Fig. 2:**
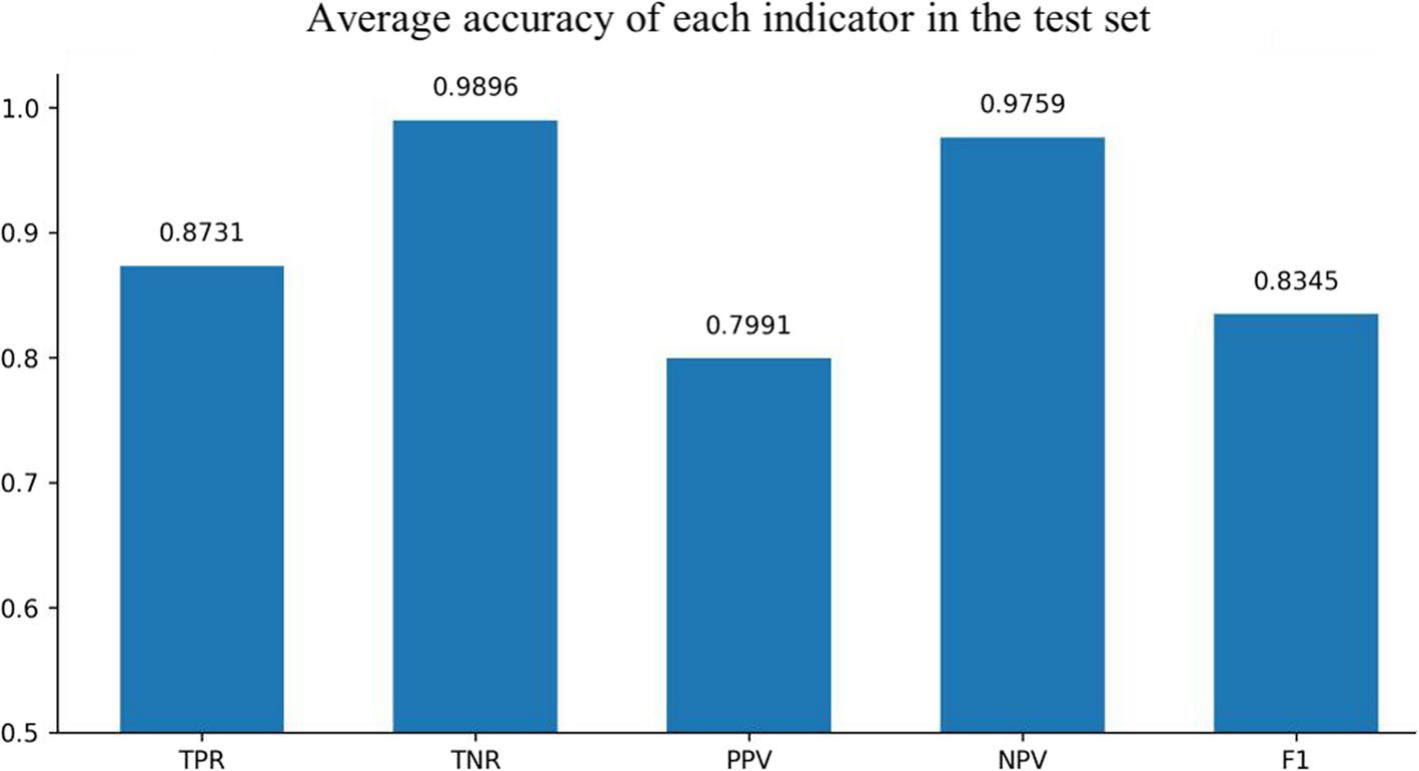
Experimental results of CNN solely

**Fig. 3:**
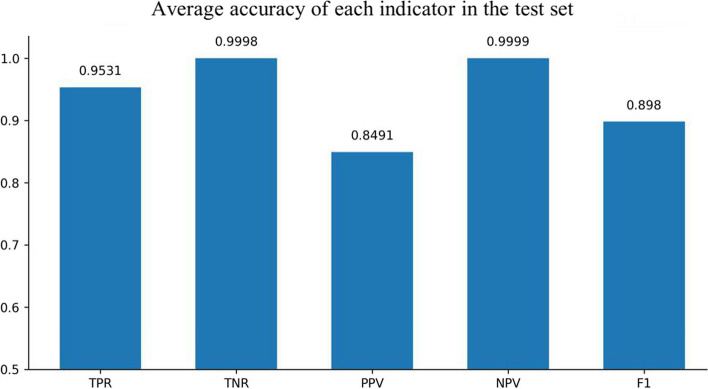
Experimental results of GRU solely

**Fig. 4:**
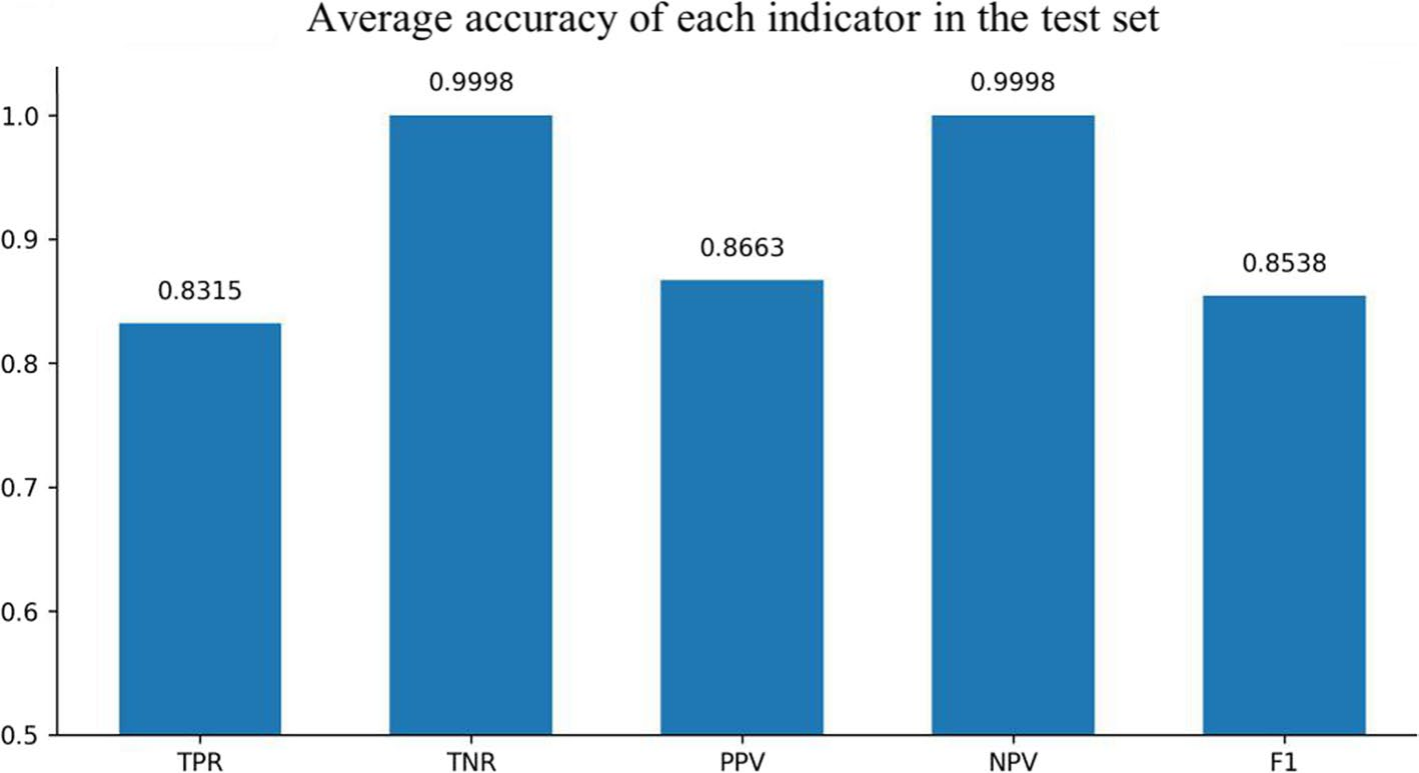
Experimental results of CNN and GRU

**Fig. 5:**
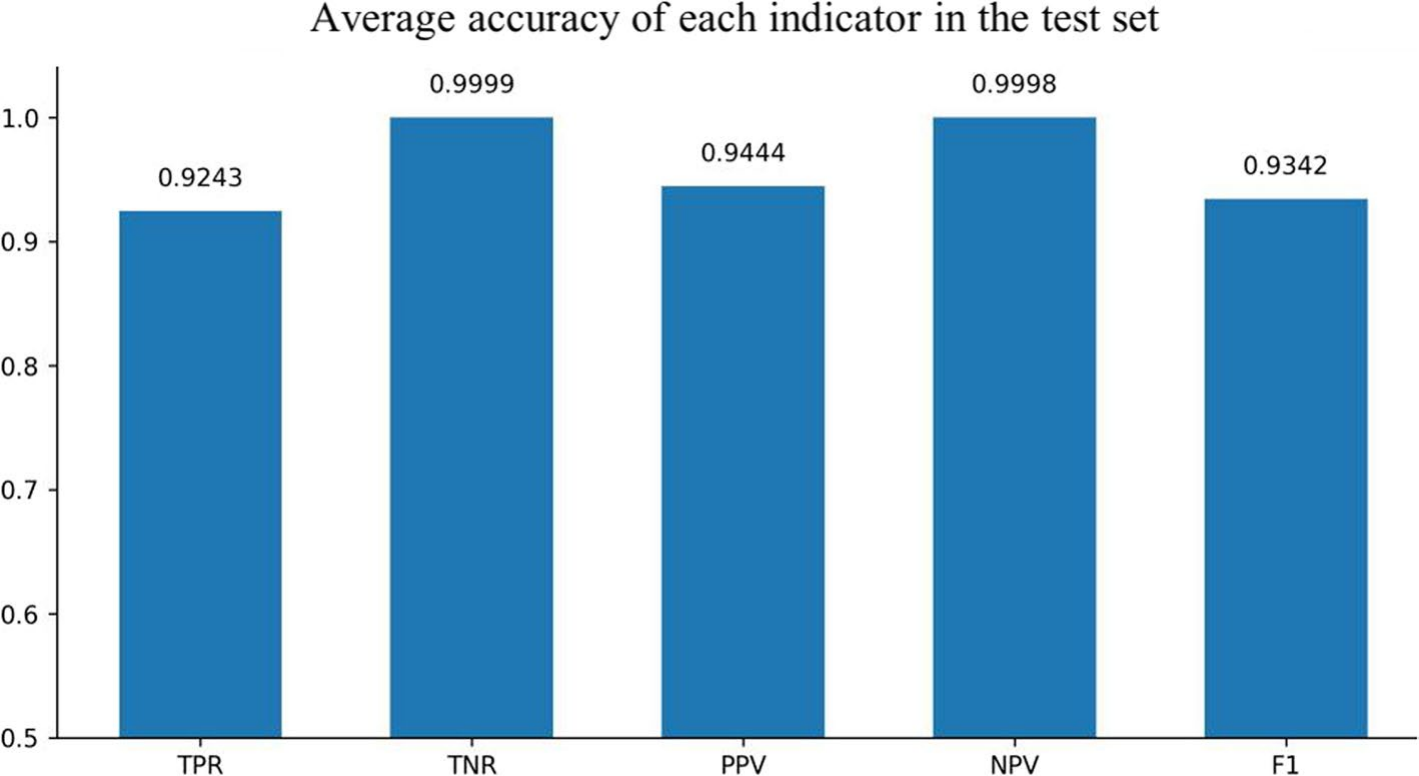
Experimental results of Resnet and GRU

Four architectures were used to train absence seizure waves in this experiment. The first model was a CNN model without Resnet and bidirectional timing GRU. The second model contained only GRU. The third model was a CNN without Resnet but it contained GRU. The fourth model contained Resnet and bidirectional timing GRU. The models trained 100 epochs, and found the best model to test the test set. The test results are shown in Figs. 2, 3, 4 and 5.

The comparative experimental results showed that the model with residual network structure had a higher sensitivity and a positive prediction value. The absence seizures waves trained data only accounted for 1.08% of the total data, and the proportion of positive and negative samples was seriously unbalanced. Since the seizure waveform of different people were not completely the same, the model added with equilibrium processing could better learn the positive sample information of different data and increase the prediction accuracy.

## Discussion

This paper proposes a method based on deep residual network and bidirectional temporal GRU to predict absence epileptic seizure waves. In the preprocessing stage, we used short-time Fourier transform to transform the data from time-domain features to time-frequency-domain features, and removed irrelevant frequency-domain segments through a band-pass filter information.

Before the data entered the neural network model, the original data of EEG was normalized to reduce the influence of data collected by different devices. The channel information obtained by different EEG signals acquisition devices was not completely consistent, we selected 19 representative channel data, namely “fp1”, “fp2”, “f3”, “f4”, “c3”, “c4”, “p3”, “p4”, “o1”, “o2”, “f7”, “f8”, “t3”, “t4”, “t5”, “t6”, “fz”, “cz”, “pz”. By changing the number of convolution kernels, CNN can effectively transform the input information from low dimension to high dimension. In order to better obtain the high-dimension information of absence seizure waves in various age groups, a deep residual network module was designed. Compared with ordinary convolutional modules, residual networks are characterized by being easy to optimize and able to increase the accuracy by adding considerable depth. The internal residual block uses skip connections to alleviate the gradient vanishing problem caused by increasing depth in deep neural networks [14–16]. Doctors judge the absence seizure waveform by analyzing the EEG data over a period of time, and then combining the characteristics of the front and back waveform. In order to make the network model learn the relational features between the sequences before and after, the GRU [17] module was designed. The GRU combines the information of the sequence before and after, and fully integrates the EEG signals of the adjacent information, which improves the accuracy of the time detection of seizures.

In order to compare the performance of the algorithm, the Support Vector Machine (SVM) and the Gradient Boosting Decision Tree (GBDT) algorithms were added to the manuscript for comparison. The test results show that the test accuracy of the two models are 0.75 and 0.84, respectively. The accuracy rate is lower than the algorithm studied in this paper. In order to verify the robust of the algorithm, we used 500 new EEG data set to test the model. The data set contains 432 normal EEG signals and 68 absent brain EEG signals. The test results show that the accuracy rate of EEG signals of normal people is 99.6%, and the accuracy rate of data including brain waves of absence is 93.4%.

Real clinical data was acquired and analyzed, primarily EEG signals from 4 weeks to 15 years of age. For infants, doctors need to combine EEG and video to diagnose the type of epilepsy. In the training process of the neural network model, strategies such as dropout and L2 regularization were used to alleviate the overfitting problem. The adaptive learning rate algorithm was used for training back propagation optimization, and the focal equalization processing method was used to process the unbalanced data, which effectively improved the performance of the model, and the overall accuracy rate reached 92%. Compared with the recent literature, we took some traditional EEG signals analysis methods as the preprocessing part, extract the data at high dimension through CNN, built a variety of models for experimental comparison to obtain an optimal network model, and made predictions, then a high accuracy rate was obtained.

Through the research in this paper, the time of EEG absence seizure waves can be quickly and accurately located, reducing the time for doctors to view EEG. It only takes 10 sec to predict a four-hour-long EEG and achieve a high precision of 92%. Doctors can make rapid and accurate judgments of absence epilepsy based on the detection results of absence seizures waves and combine with video analysis of corresponding points. Only EEGs were analyzed in this research, videos weren't included. Future work is planned to perform multi-model analysis through EEG and video, further improve accuracy and be clinically helpful to doctors.

## Conclusions

In this manuscript, more than a thousand of cases from Kunming Children’s Hospital were analyzed and studied. Convolutional networks can extract absence seizure waves to identify features, and can also be used to infer absence epilepsy patterns in EEG. After GRU processing, a better inference accuracy will be obtained. In addition, the reasoning speed is also at a relatively fast speed level. This method can be effectively used in EEG signals software to provide reference for doctors in EEG analysis and save “EEG reading time”, which is of great practical value.

## Data Availability

All data generated or analyzed in this study are included in this manuscript.

## Abbreviations

BPF: Band-pass filter
CNN: Convolutional Neural Networks
DWT: Wavelet transform
EEG: Electroencephalogram
GBDT: Gradient Boosting Decision Tree
GRU: Gate Recurrent Unit
NPV: Negative predictive value
PCA: Principal component analysis
PPV: Positive predictive value
STFT: Short-time Fourier transform
SVM: Support Vector Machine
TAS: Typical absence seizures
TNR: True negative rate
TPR: True positive rate

## References

[1] Elaine K. Understanding Seizures: Epilepsy Foundation; 2019. https://www.epilepsy.com/what-is-epilepsy/understanding-seizures Accessed 3 Dec 2022

[2] Fisher RS, Cross JH, Jacqueline A, Higurashi N, et al. Operational classification of seizure types by the international league against epilepsy: position paper of the ILAE Commission for Classification and Terminology. Epilepsia. 2017;58(4):522–30. 28276060 10.1111/epi.13670

[3] Wolpaw JR, Ramoser H, McFarland DJ, Pfurtscheller G. EEG-based communication: improved accuracy by response verification. IEEE Trans Rehab Eng. 1998;6(3):326–33. 10.1109/86.712231 9749910

[4] Iasemidis LD, Sackellares JC, Zaveri HP, Williams WJ. Phase space topography and the Lyapunov exponent of electrocorticograms in partial seizures. Brain Topogr. 1990;5(2):187–201. 10.1007/BF01140588 2116818

[5] Lehnertz K, Elger CE. Spatio-temporal dynamics of the primary epileptogenic area in temporal lobe epilepsy characterized by neuronal complexity loss. Electroencephalogr Clin Neurophysiol. 1995;95(2):108–17. 7649002 10.1016/0013-4694(95)00071-6

[6] Blanco S, Rosso OA, Salgado P. Applying time-frequency analysis to seizure EEG activity. IEEE Eng Med Biol Mag. 1997;16(1):64–71. 9058584 10.1109/51.566156

[7] Khan YU, Gotman J. Wavelet based automatic seizure detection in intracerebral electroencephalogram author links open overlay panel. Clin Neurophysiol. 2003;114(5):898–908. 12738437 10.1016/s1388-2457(03)00035-x

[8] Sartorettoa F, Mario E. Automatic detection of epileptiform activity by single-level wavelet analysis. Clin Neurophysiol. 1999;110(2):239–49. 10210613 10.1016/s0013-4694(98)00116-3

[9] Thomasson N, Heppner TJ, et al. Recurrence quantification in epileptic EEGs. Phys Lett A. 2001;279(1–2):94–101.

[10] Antoniades A, Spyrou L, Martin-Lopez D, et al. Detection of Interictal discharges with convolutional neural networks using discrete ordered multichannel intracranial EEG. IEEE Trans Neural Syst Rehab Eng. 2017;25(12):2285–94. 10.1109/TNSRE.2017.2755770 28952945

[11] Amin HU, Yusoff MZ, Ahmad RF. A novel approach based on wavelet analysis and arithmetic coding for automated detection and diagnosis of epileptic seizure in EEG signals using machine learning techniques. Biomed Signal Process Control. 2020;56:101707.

[12] Giannakakis G, Tsekos N, Giannakaki K, Michalopoulos K, Zervakis M, editors. Seizure detection using common spatial patterns and classification techniques. IEEE 19th international conference on bioinformatics and bioengineering (BIBE); 2019.

[13] Zhang Y, Wang Y, Zhou G, Jin J, Wang B, Wang X, et al. Multi-kernel extreme learning machine for EEG classification in brain-computer interfaces. Expert Syst Appl. 2018;96:302–10.

[14] He K, Zhang XY, Ren SQ, Sun J. Deep residual learning for image recognition. In: Proceedings of the IEEE conference on computer vision and pattern recognition; 2016. p. 770–8.

[15] Brigo F, Igwe SC, Lattanzi S. Ethosuximide, sodium valproate or lamotrigine for absence seizures in children and adolescents. Cochrane Database Syst Rev. 2021;1(1):Cd003032. 10.1002/14651858.CD003032.pub5 PMC8095003 33475151

[16] Wei XY, Zhou L, Chen ZY, et al. Automatic seizure detection using three-dimensional CNN based on multi-channel EEG. BMC Med Inform Decis Mak. 2018;18(12):71–80. 30526571 10.1186/s12911-018-0693-8 PMC6284363

[17] Zhou R, Hu B, Xu Q. Lithology classification system for well logging based on bidirectional gated recurrent unit. 2021 4th International conference on artificial intelligence and big data (ICAIBD); 2021. p. 599–603.

