# Correction: Detection method of absence seizures based on Resnet and bidirectional GRU

**DOI:** 10.1186/s42494-023-00119-2

**Published:** 2023-03-14

**Authors:** Lijun Li, Hengxing Zhang, Xiaomei Liu, Jie Li, Lei Li, Dan Liu, Jieqing Min, Ping Zhu, Huan Xia, Shangkun Wang, Li Wang

**Affiliations:** 1 Kunming children’s hospital, Kunming, 650000 China; 2 Zhengzhou Zoneyet Technology Corp.,Ltd, Zhengzhou, 450000 China

**Correction: Acta Epileptologica 5, 7 (2023)**

**https://doi.org/10.1186/s42494-022-00117-w**

Following publication of the original article [1], the editorial office reported that the Competing Interests “not applicable” should be updated.

The updated Competing Interests should read: Author HZ, JL and DL are employees of Zhengzhou Zoneyet Technology Corp.Ltd, and JL is the shareholder. The ongoing patents relating to the content of the manuscript are CN202210949695.2 and CN202210885566.1. Zhengzhou Zoneyet Technology Corp.Ltd provides study materials, writing and technical support.

The original article [1] has been updated.
